# Relationship between serum levels of triglycerides and vascular inflammation, measured as COX-2, in arteries from diabetic patients: a translational study

**DOI:** 10.1186/1476-511X-12-62

**Published:** 2013-05-03

**Authors:** Antonio Gordillo-Moscoso, Emilio Ruiz, Manuel Carnero, Fernando Reguillo, Enrique Rodriguez, Teresa Tejerina, Santiago Redondo

**Affiliations:** 1 Department of Pharmacology, School of Medicine, Universidad Complutense, Madrid, Spain; 2 Service of Cardiac Surgery, Hospital Clínico San Carlos, Madrid, Spain

**Keywords:** Human arteries, Lipid profile, COX-2, Diabetes mellitus

## Abstract

**Background:**

Inflammation is a common feature in the majority of cardiovascular disease, including Diabetes Mellitus (DM). Levels of pro-inflammatory markers have been found in increasing levels in serum from diabetic patients (DP). Moreover, levels of Cyclooxygenase-2 (COX-2) are increased in coronary arteries from DP.

**Methods:**

Through a cross-sectional design, patients who underwent CABG were recruited. Vascular smooth muscle cells (VSMC) were cultured and COX-2 was measured by western blot. Biochemical and clinical data were collected from the medical record and by blood testing. COX-2 expression was analyzed in internal mammary artery cross-sections by confocal microscopy. Eventually, PGI_2_ and PGE_2_ were assessed from VSMC conditioned media by ELISA.

**Results:**

Only a high glucose concentration, but a physiological concentration of triglycerides exposure of cultured human VSMC derived from non-diabetic patients increased COX-2 expression .Diabetic patients showed increasing serum levels of glucose, Hb1_ac_ and triglycerides. The bivariate analysis of the variables showed that triglycerides was positively correlated with the expression of COX-2 in internal mammary arteries from patients (r^2^ = 0.214, P < 0.04).

**Conclusions:**

We conclude that is not the glucose blood levels but the triglicerydes leves what increases the expression of COX-2 in arteries from DP.

## Introduction

Over the past two decades increases in obesity, due to high caloric intakes and immobilizing technologies, has led to a surge in type 2 diabetes. In obesity elevated circulating fatty acids set-off a pro-inflammatory cascade, a common feature with diabetes mellitus. Epidemiological studies show that the prevalence of Diabetes Mellitus (DM) has increased worldwide in the last few years [1,2]. Diabetes Mellitus confers a 2- to 4-fold increase in cardiovascular risk compared with the general population [3]. Type 2 DM usually coexists with other risk factors such as hypertension and hyperlipidemia. This scenario leads to vascular complications which are the main cause of morbidity and mortality in these patients. Along with increasing levels of blood glucose, diabetic patients also exhibit dyslipemia. Dyslipemia characteristically consists of decreased levels of high-density lipoproteins (HDL) cholesterol, elevated levels of low-density lipoprotein (LDL) cholesterol and elevated levels of triglycerides (TG) [4-6]. Regarding the risk factors responsible for the evolution of atherosclerosis in diabetic patients, Bierman [7] estimated that typical risk factors including smoking, cholesterol and blood pressure can account for no more that 25-30% of excess cardiovascular risk factor in diabetic patients. This suggests that other factors might play a key role in the progression of atherosclerosis in diabetes. In this context, cardiovascular disease and diabetes are both associated with elevated levels of inflammatory biomarkers including cytokines and C-reactive protein (CRP) [8]. Another inflammatory protein involved in atherosclerosis is cyclooxygenase-2. Cyclooxygenases-1 and −2 (COX-1 and COX-2), which are the key enzymes in prostaglandin biosynthesis, are thought to be important in maintaining the normal physiological function and certain pathological processes. The inducible form of COX, named COX-2, is not expressed under physiological conditions but it can be rapidly expressed under certain circumstances [9]. COX-2 is a rate-limiting enzyme that catalyzes the conversion of free arachidonic acid into prostaglandin H2, the first step in the biosynthesis of prostaglandin and thromboxane. A recent paper has shown that COX-2 is highly expressed in vascular smooth muscle cells (VSMC) in a type 2 diabetic mouse model [10] and in coronary arteries from diabetic patients [11]. Moreover, results from our group show that COX-2 expression is higher in internal mammary arteries from diabetic patients and this is closely related to increased expression of the antiapoptotic protein Bcl-2 [12]. This mediates an increased proliferation/apoptosis ratio in VSMC from diabetic patients and a subsequent pathologic vascular remodeling [13].

This enzyme might be responsible, at least in part, for some of the changes in vascular reactivity that occur in arteries from diabetic patients [11]. Although high glucose conditions have been involved in the expression of COX-2 in VSMC in vitro [14] there is no further information about which serum component, if any, might trigger the differential expression of COX-2 in arteries from diabetic patients. Therefore, we aim to determine which serum biomarkers could explain the elevation in COX-2 expression in arteries from type 2 diabetic patients. Identification of such serological markers may reveal new approaches to the prevention of cardiovascular complications in diabetic and pre-diabetic patients.

## Results

### Characteristics of the patients

**Figure 1:**
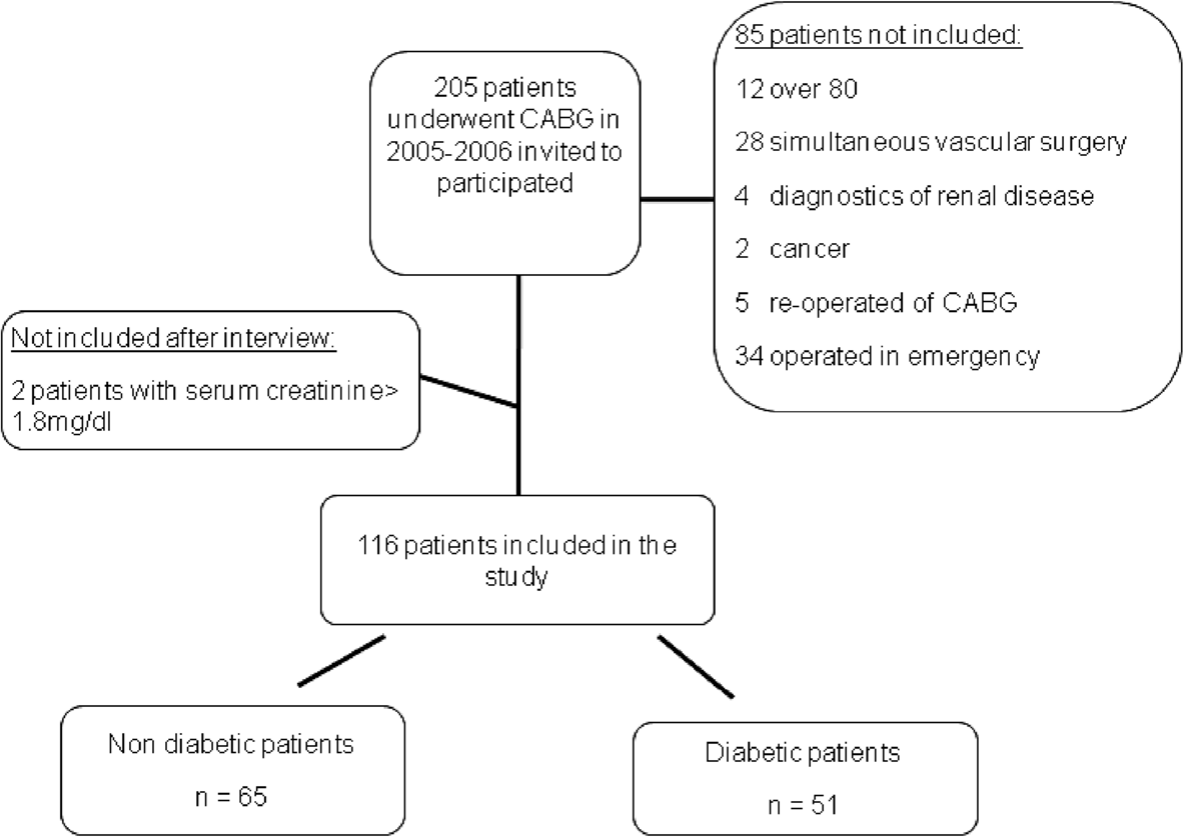
**Flow diagram of the patients.** Patients were recruited as stated in Methods.

A group of 116 atherosclerotic patients were recruited from those undergoing CABG in the Service of Cardiac Surgery (Hospital Clínico San Carlos, Madrid). A flow diagram of the patients is shown in Figure 1.

Of these patients, 15% were women and 33% smokers and the age was 64.7±+9 years old (ranged from 37 to 80 years old). The patients shared the classical comorbidities such as hypertension (58% of the patients), hyperlipidemia (59% of the patients) and Diabetes Mellitus (44%) as shown in Table 1. In terms of biochemical characteristics, glucose and percentage of Hb1ac were higher in diabetic patients (DP) compared with non-diabetic patients (non-DP). We also found that triglycerides were elevated in DP whereas LDL was lower (Table 2).

**Table 1:** Clinical characteristics of the patients studied

**Variable**	**Complete group**	**Non- diabetic**	**Diabetic**	**P**
Number of patients	116	65	51	-
Age (years)	64.7 ± 9	63.4 ± 9.5	66.4 ± 8.2	0.07 *
Women (%)	17 (15%)	8 (12%)	9 (18%)	0.42 ^†^
Body Mass Index	27.3 ± 3.4	27 ± 3.2	27.6 ± 3.7	0.37 *
Smoking	38 (33%)	25 (38%)	13 (25%)	0.13 ^†^
Hypertensives	64 (55%)	18 (35%)	33 (56%)	0.06 ^†^
Hyperlipidemics	69 (59%)	37 (57%)	32 (63%)	0.53 ^†^
Previous AMI (<30days)	20 (17%)	14 (21%)	6 (12%)	0.17 ^†^
Previous AMI (31–90 days)	9 (8%)	2 (4%)	6 (12%)	0.13 ^‡^
Previous AMI (>90days)	37 (32%)	16 (25%)	21 (41%)	0.05 ^†^

BMI = body mass index. AMI = acute myocardial infarction. ^*^ = Student T-test, ^†^ = Chi-squared, ^‡^ = Fisher test.

**Table 2:** Biochemical characteristics of the patients studied

**Variable**	**Complete group**	**Non-diabetic**	**Diabetic**	**P**
Glucose (mg/dL) ^¶^	110 ± 44	93 ± 19	136 ± 50	< 0.001 *
Hb1_ac_ (%) ^¶^	6.4 ± 1.2	5.6 ± 0.4	7.4 ± 1.1	< 0.001 *
Total cholesterol (mg/dL) ^¶^	154 ± 47	158 ± 51	148 ± 40	0.221 ^§^
HDL-cholesterol (mg/dL) ^¶^	41 ± 12	42 ± 14	39 ± 10	0.238 ^§^
LDL-cholesterol (mg/dL) ^¶^	82 ± 37	89 ± 40	73 ± 32	0.019 ^§^
non-HDL cholesterol (mg/dL) ^¶^	113 ± 41	116 ± 43	116 ± 38	0.230 ^§^
Triglycerides (mg/dL) ^¶^	137 ± 68	125 ± 42	154 ± 87	0.005 ^§^
Creatinine (mg/dL) ^¶^	1.08 ± 0.2	1.08 ± 0.2	1.08 ± 0.2	0.530 ^§^
Creatinine (mg/dL) ^¶^	1.08 ± 0.2	1.08 ± 0.2	1.08 ± 0.2	0.530 ^§^
Fibrinógen (mg/dL)	453 ± 102	455 ± 110	451 ± 93	0.841 ^§^
Homocysteine (μmol/L)	11 ± 2.9	11 ± 1.5	11 ± 2.9	0.499 ^¤^
Apoprotein A (mg/dL)	116 ± 25.2	118 ± 28.1	114 ± 19.3	0.551 ^¤^
C-reactive protein (mg/mL)	3.6 ± 3.8	3.3 ± 3.4	3.6 ± 3.8	0.880^¤^
Interleukin-6 (pg/mL)	9.3 ± 3.1	9.46 ± 2.8	9.21 ± 3.3	0.750^¤^
TNF-α (pg/mL)	6.2 ± 2.2	6.3 ± 2.2	6.1 ± 2.1	0.525^¤^
MMP-9 (ng/mL)	301 ± 300	458 ± 186	260 ± 397	0.103^¤^

^¶^ = ? armonic mean, ^§^ = Student T-test, * = Welch test (Beherens-Fisher), ^¤^ = U test Mann–Whitney-Wilcoxon), mad = media absolute deviation.

### Expression of COX-2 in VSMC induced by glucose or triglycerides

**Figure 2:**
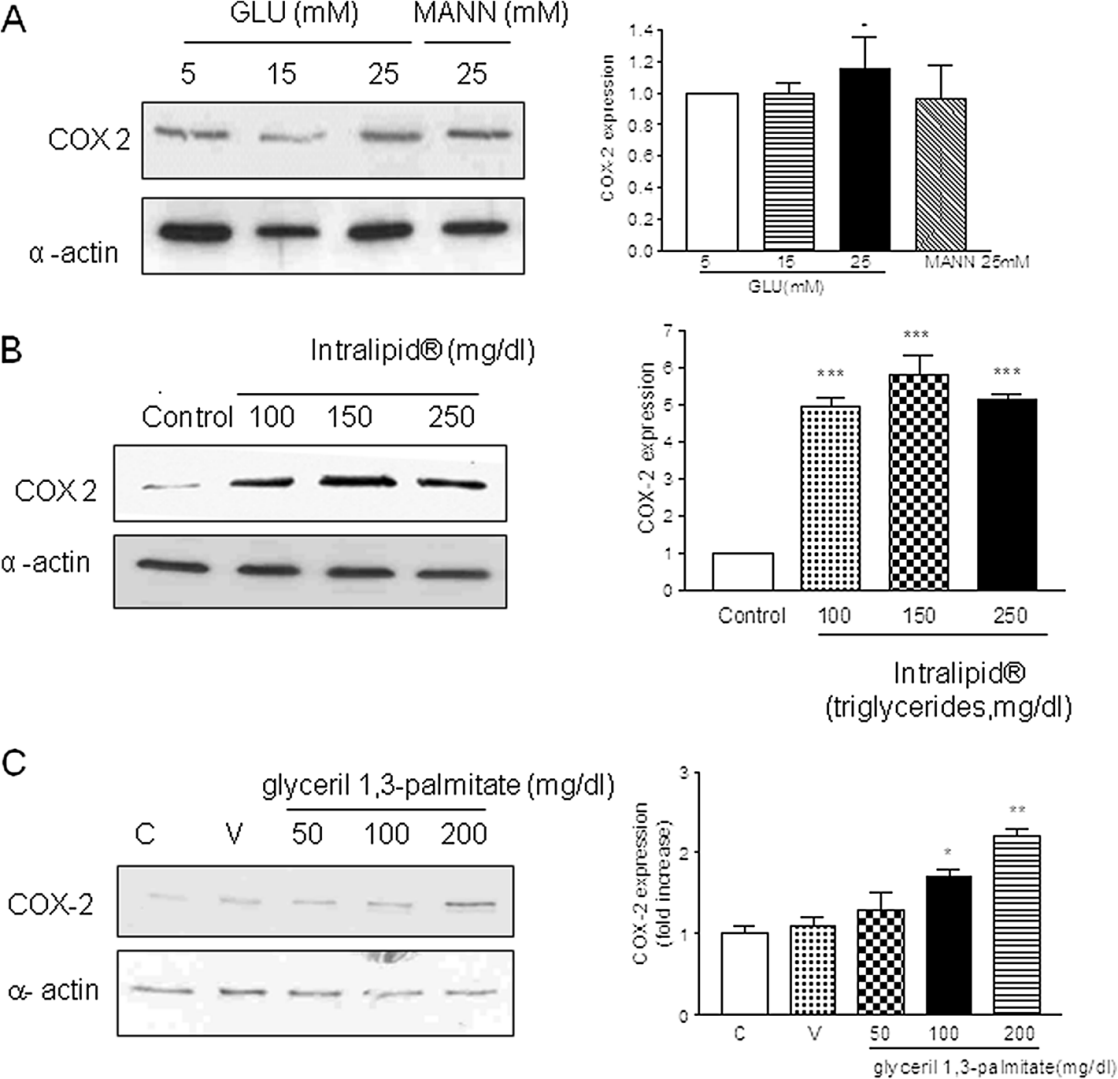
**Expression of COX-2 in human vascular smooth muscle cells.** Panel **A**: effect of glucose. Left panel: Western blotting shows the expression of COX-2 in non diabetic VSMC incubated with increasing concentrations of glucose. Right panel: Bar graph shows the mean±SEM of n = 4 patients, *P < 0.05 with respect to 5 mM glucose. Panel **B**: effect of triglycerides. Left panel: Western blotting shows the expression of COX-2 in non diabetic VSMC incubated with increasing concentrations of triglycerides. Right panel: Bar graph shows the mean±SEM of n = 4 patients, ***P < 0.001 with respect to control (vehicle, glycerol). Panel **C**: Effects of glyceril palmytate as a pure source of triglycerides.

We analyzed in vitro whether increasing concentrations of glucose or triglycerides induced the expression of COX-2. Since basal expression of COX-2 in cells from DP was already high, we used VSMCV from non-diabetic subjects. Human VSMC from non diabetic patients treated with increasing concentrations of glucose (5-25 mM) showed COX-2 expression was very slightly increased with the highest concentration of glucose used (25 mM) (Figure 2, Panel A). On the other hand, treatment of VSMC from non diabetic patients with an increasing concentration of triglycerides (100-250 mg/dL) showed a marked increase in the expression of COX-2 as shown in Figure 2, Panel B.

### Relationship between serum parameters and COX-2 expression in patients

**Figure 3:**
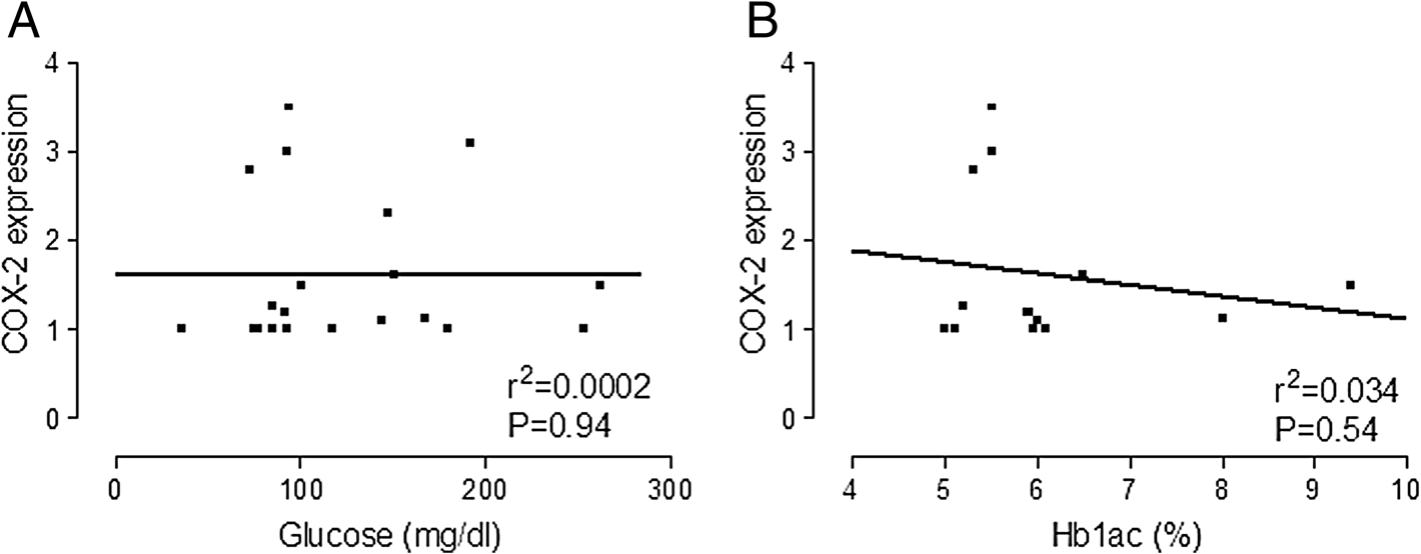
**Bivariate correlation between serum glucose (panel A) or Hb1ac (panel B) and COX-2 expression COX-2 expression was quantified in the media layer of internal mammary arteries from DP and non-DP by confocal microscopy (n = 34 patients).** Analysis was performed by Pearson correlation.

**Figure 4:**
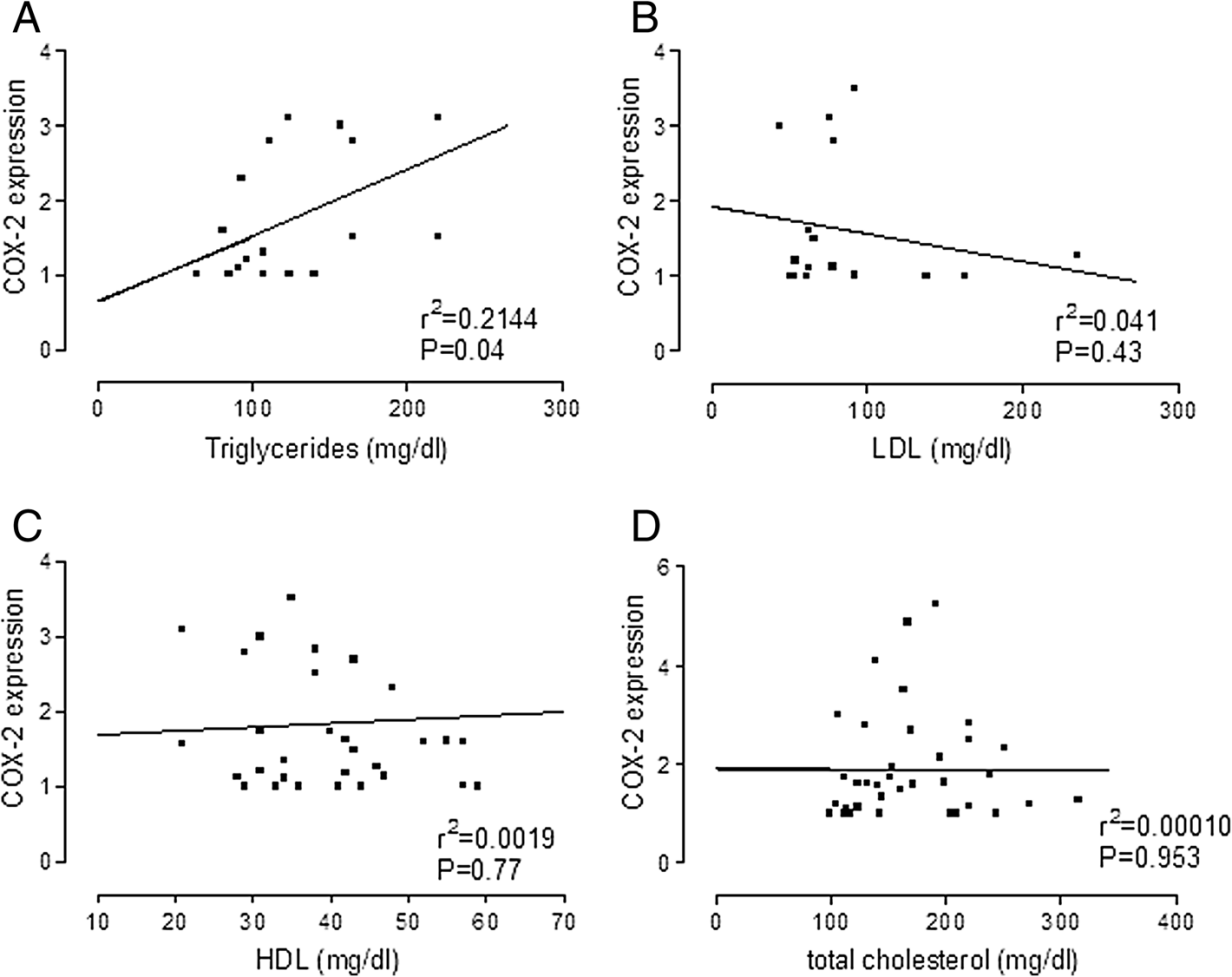
**Bivariate correlation between the lipid profile and COX-2 expression in internal mammary arteries.** COX-2 expression was quantified in the media layer of internal mammary arteries from DP and non-DP by confocal microscopy (n = 34 patients). Analysis was performed by Pearson correlation.

Some authors have shown that the expression of COX-2 in VSMC in culture was induced by high levels of glucose [14]. However, it is not clear whether serum levels of glucose are related with the levels of COX-2 expression. We studied the vascular expression of the inflammatory protein COX-2 in cross-sections of internal mammary arteries from DP and non-DP analyzed by confocal microscopy and analyzed the correlation between serum glucose or Hb1_ac_ and COX-2 expression in arteries. As shown in Figure 3, there was no statistically significant correlation between glucose or Hb1_ac_ and COX-2 expression. However, in terms of the lipid profile, we found a good correlation between serum levels of triglycerides and COX-2 expression (Figure 4, panel A), but not with LDL (Figure 4, panel B), HDL (Figure 4, panel C) or total colesterol (Figure 4, panel D). In another set of experiments, we measured the expression of the antioxidant protein HO-1 in internal mammary arteries from DP and non-DP by confocal microscopy. However, a statistically signigicant difference was not found (2.027 ± 0.2352 vs. 2.140 ± 0.1039 arbitrary units, p = 0.7173).

### Analysis of prostaglandin production and MMP-9 levels

**Figure 5:**
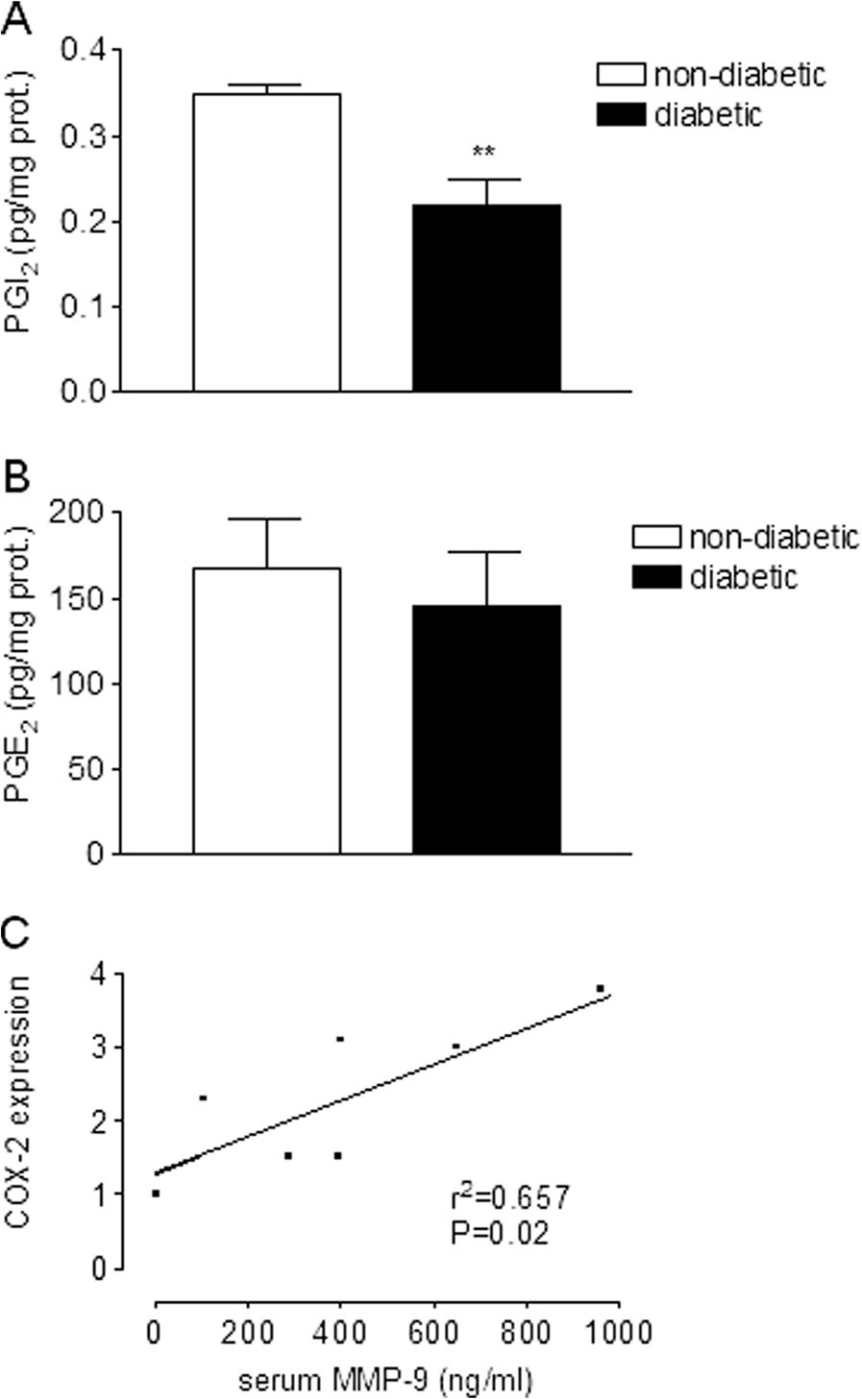
**Consequence of COX-2 expression.** Basal release of prostacyclin (Panel **A**) or PGE_2_ (Panel **B**) in VSMC from DP and non-DP. Bar graph shows the mean±SEM of n = 4 patients, **P < 0.01. Panel **C**: Bivariate correlation between serum MMP-9 levels and COX-2 expression in internal mammary arteries. Analysis was performed by Pearson correlation.

To study the consequences of COX-2 expression, we determined the production of PGI_2_ and PGE_2_ in human VSMC from DP and non-DP. As shown in Figure 5, Panel A, the basal release of PGI_2_ into the cellular medium was decreased in vascular smooth muscle cells isolated from diabetic patients compared with non-diabetic. However, the basal production of PGE_2_ was similar in human vascular smooth muscle cells from DP compared with non-DP (Figure 5, Panel B). On the other hand, since production of PGE_2_ has been associated with increasing levels of MMP-9, we studied the correlation between COX-2 expression in the vasculature with the serum levels of MMP-9. Figure 5, Panel C shows that COX-2 expression correlates with the serum MMP-9 (r^2^ = 0.657, P = 0.02).

## Discussion

In the present work we have found that the inflammatory enzyme COX-2 was highly expressed in internal mammary arteries from diabetic patients. Moreover, since our aim was to evaluate which serum biomarker(s) was (were) responsible for COX-2 expression, we performed a translational study in which we recruited a group of 116 atherosclerotic patients with or without DM as a risk factor. Data from this study showed that DP had increasing levels of glucose and Hb1_ac_, as expected, but also triglycerides (TG). From this, we studied the correlation of these markers with the vascular expression of COX-2 by a bivariate analysis and we found that TG levels correlated with the expression of COX-2.

Some authors have shown previously that high levels of glucose induced COX-2 expression in several cell types. High glucose-induced COX-2 expression in monocytes is regulated by NFk-B, protein kinase-C and p38 MAPK signalling pathways [15]. Protein kinase-C has also been involved in COX-2 expression in rat vascular smooth muscle cells in culture [16]. However, a comprehensive analysis of the intracellular mechanism underlying COX-2 expression in human vascular smooth muscle cells is out of the scope of this manuscript.

We evaluated in human vascular smooth muscle cells in culture the effect of different concentrations of glucose in the cellular medium. We found that glucose 25 mM slightly but significantly increased the expression of COX-2 in cells from non-DP. Although sporadically glucose levels in DP may reach the concentration of 25 mM, the glucose levels in blood from our diabetic patients were in a range of 8-10 mM. In fact, the bivariate analysis of glucose (or Hb1_ac_) versus COX-2 expression showed no correlation, which indicates that the expression of COX-2 in arteries from DP should be related with other factors. Another possibility was that the expression of COX-2 could be related to changes in the lipid profile of our patients. As shown in Figure 3, the levels of triglycerides positively correlated with the expression of COX-2. This fact indicates that it is the lipid profile rather than glycaemia which might regulate the expression of COX-2 in diabetic patients. This, along with the fact that levels of triglycerides were higher in diabetic patients, leads to the conclusion that elevated triglyceride levels in diabetic patients, but not glucose, is responsible for the overexpression of COX-2 in arteries from diabetic patients. To corroborate this, when human VSMC from non-DP were treated with different concentrations of triglycerides we found that, unlike glucose, treatment with triglycerides induced COX-2 expression in cells from ND patients.

Dislipemia, associated with high levels of triglycerides and low concentrations of HDL contributes to a proinflammatory state [17]. In this context, the adipose tissue, in addition to storing calories as triglycerides, also secretes a large variety of pro-inflammatory proteins [18] which contributes to cardiovascular disorders.

The consequences of COX-2 expression in atherosclerotic vessels are not fully understood. The induction of COX-2 *in vivo* is generally associated with deletereous responses. However, in the presence of endothelial dysfunction (i.e. in diabetes) the local induction of COX-2 in the underlying smooth muscle cells may compensate for the reduced thrombo-resistance of that section of the vessel and may also compensate for the decrease in nitric oxide-dependent vasorelaxation observed in diabetic arteries [19]. Thus, endogenous PGI_2_ release as a result of COX-2 expression is considered beneficial in the cardiovascular system since it decreases VSMC proliferation [20], cholesterol accumulation and platelet activation and increases vasodilation [21,22]. Interestingly, PGI_2_ synthesis from human aorta samples decreases as a function of progressing atherosclerotic lesion, whereas PGE_2_ increases in parallel [23]. PGE_2_ is a proatherogenic eicosanoid when released in advanced atherosclerotic plaques since it may induce the release of metalloproteinases (MMP) such MMP-2 and MMP-9, enzymes capable of degrading all macromolecular constituents of the extracellular matrix [24] and thus participate in atherothrombosis. In this work, we evaluated the release of basal PGI_2_ and PGE_2_ in human VSMC isolated from DP and non-DP. We found that, although the basal levels of PGE_2_ were similar in both groups of patients, the release of PGI_2_ decreased in cells from diabetic patients. Some postulations may be made about the surprising fact the fact that PGI_2_ levels in cells from diabetic patients was lower than in non-diabetic ones. One of the most outstanding candidates for PGI2 inhibition in the diabetic scenario is the peroxynitrite oxygen reactive form (ONOO^-^), which has been shown to perform a selective nitration PGI_2_ synthase in *in vitro* models of diabetes and therefore inhibit PGI2 synthesis [25]. This increase in ONOO^-^ is thought to take place by means of eNOS uncoupling in diabetes [26], which may be related to a decreased eNOS expression in the internal mammary arteries of diabetic patients who underwent by-pass surgery. Moreover, the analysis of COX-2 and MMP-9 indicated a correlation between these proteins. The latter might indicate that, persistent overexpression of COX-2 in diabetic patients might lead to a deleterious effect.

According to our results, it is intriguing to systematically assess plasma and urine levels of eicosanoids such as LTs and TXs, plasma and RBC membrane levels of antioxidants such as SOD, catalase and glutathione as well as plasma levels of NO in DP and non-DP undergoing CABG surgery. This could help to broaden our knowledge about how diabetes affects the balance among lipids, inflammation, eicosanoids, oxidative stress and subsequent endothelial funcition and will be the goal of coming research efforts.

## Conclusions

In conclusion, this work describes for the first time that vascular inflammation in diabetic patients is determined by changes in the lipid profile, rather than by glycaemia. This might have important clinical implications in the way that diabetic patients need to be treated to prevent cardiosvacular complications originated by the inflammatory process.

## Methods

### Materials

All reagents were obtained from Sigma (Spain) unless otherwise stated.

### Patients

A group of patients was recruited from those undergoing coronary artery bypass graft surgery at the Cardiac Surgery Service (Hospital Clinico San Carlos, Madrid, Spain). Diabetes Mellitus was defined following the criteria established by the ADA (American Diabetes Association) [27] as fasting serum glucose concentration ≥126 mg/dl and use of antidiabetic oral drugs or insulin. Patient data included: age, gender, active smoker, obesity, total cholesterol, cholesterol LDL, cholesterol HDL, triglycerides, glucose and blood pressure. Exclusion criteria of the patients included patients older than 80 years of age, pathologies that affect the inflammatory status (renal failure, liver disease, etc.) and cancer. Internal mammary arteries were collected by the surgeons during the surgical procedure, labeled and used within the next few minutes after the operations. The study was conducted according to the Declaration of Helsinki and we obtained informed consent from all subjects before sampling took place. The study was approved by the local Ethical Committee (Hospital Clinico San Carlos, Madrid, Spain). From all included patients we had access to the clinical report and blood sample. However, for surgical limitations, a proper internal mammary artery segment for further confocal microscopy analysis or cell cultures experiments could not be obtained from all CABG patients. Thus, we performed the experiments under controlled conditions with the maximal number of samples we could obtain from our clinical group.

### Analysis of blood samples

Serum samples were collected from each patient by the Service of Clinical Analysis (Hospital Clínico San Carlos, Madrid) before the operations took place. Typical biochemical biomarkers (cholesterol, glucose, triglycerides, LDL, HDL, etc.) were analyzed in the Service of Clinical Analysis. Serum samples for cytokines profile were prepared, frozen and stored at −70°C until the assay. Levels of C-reactive protein (CRP), interleukin-6 (IL-6) matrix metalloproteinase-9 (MMP-9), and CD40L were measured by enzyme-linked immunosorbent assay (ELISA) (Quantikine, R&D System, Madrid, Spain).

### Cell cultures

Human internal mammary artery vascular smooth muscle cells (VSMC) were cultured from explants in RPMI (Life Technologies, Barcelona, Spain) containing 10% fetal calf serum (FCS). The cells exhibited typical “hill and valley” smooth muscle morphology by phase contrast microscopy and the cultures were stained positively with a monoclonal anti-smooth α-actin antibody. Experiments were performed with VSMC between passages 3 and 5. To determine the expression of COX-2, cells were plated onto 60 mm tissue culture dishes and allowed to attach for 24 h. To analyze the effect of glucose or triglycerides on COX-2 expression, VSMC from non-diabetic patients were treated for 48h with 5–25 mM of glucose (or mannitol 25 mM) or with triglycerides (purified soya oil emulsion Intralipid©, Fresenius-Kabi, Madrid, Spain) at 100-250 mg/dL for 24 h.

### Western blotting

At the time of harvest, the cells were washed with ice-cold PBS, lysed on ice with 200 μL lysis buffer (10% glycerol, 2.3% SDS, 62.5 mM Tris–HCl, pH 6, 8 150 mM NaCl, 10 mM EDTA, 1 μg/mL leupeptin, 1 μg/mL pepstatin, 5 μg/mL chymostatin, 1 μg/mL aprotinin, 1 mM phenylmethylsulphonyl fluoride) and boiled for 5 min. Equal amounts of protein were run on 10% SDS-polyacrylamide gel electrophoresis. The proteins were then transferred to polyvinylidene difluoride (PVDF) membranes (Immobilon-P, Amersham, Madrid, Spain), and blocked overnight at 4°C in blocking solution (3% BSA in TBS-T: 25 mM Tris–HCl, 75 mM NaCl, pH = 7.4, 0.1% v/v Tween 20). For analysis of COX-2 expression, the blots were incubated overnight with agitation at 4°C in the presence of a specific mouse monoclonal anti-COX-2 antibody (Transduction Labs., Madrid, Spain) at 1:1000 in in 0.3% bovine serum albumin in TBS-T. After washing in TBS-T solution, the blots were further incubated for 1 h at room temperature with a horseradish peroxidase conjugated anti-mouse secondary antibody diluted 1:10,000 (Santa Cruz Biotechnology, CA, USA) in blocking solution. The blots were then washed 5 times in TBS-T and antibody-bound protein was visualized with Enhanced Chemiluminescence’s (ECL) kit (Amershan Biosciences, Barcelona, Spain). Smooth muscle α-actin was used as a housekeeping protein and it was determined following the same procedure as mentioned above, using a specific anti-α-actin mouse monoclonal antibody (Sigma-Aldrich, Madrid, Spain), at 1:1,000 in TBS-T.

### Immunofluorescence staining

Small segments of internal mammary arteries were fixed with 4% paraformaldehyde for 2 h, washed with PBS, immersed in PBS 0.1 mol/l + sucrose 30% at 4°C for 3 h and embedded in OCT (OCT-tissue tek, Bayer) for 30 min. Cross sections 7 μm thick were obtained (Cryostat HM500, Microm international GMBH, Dusseldorf), dried at 37°C and washed with PBS + 0.3% Tween-20 (PBS-T). Unspecific binding was blocked by incubating the samples for 1 h in 3% bovine albumin in PBS-T. Cross sections were incubated with mouse monoclonal anti-COX-2 or anti-HO-1 antibodies at 1:100 (BD Transduction Labs., Madrid, Spain) for 1 h at 37°C and after washing, with Alexa-568 goat anti-mouse secondary antibodies (Molecular Probes, USA) at 1:500 for 1 h at 37°C. Confocal microscopy images were by captured using a Leica TCS SP2 inverted microscope (objective = 63×, the excitation and emission wave length were 543 and 603 nm respectively). Semiquantitative measure of COX-2 expression was performed by using the Image J 1.33 software (NIH, USA). Data are presented as fold increase of protein expression with respect to each negative control. COX-2 expression was quantified in the media layer of internal mammary arteries from DP and non-DP by confocal microscopy (n = 34 patients). Analysis of COX-2 intensity was done by two independent researchers and the concordance analyzed with the Intraclass Correlation Coefficient. The value obtained was 0.78, within acceptable range.

### Analysis of PGE_2_ and PGI_2_ production

Release of PGE_2_ and PGI_2_ into the culture medium was determined as an index of COX-2 activity. Vascular smooth muscle cells from DP and non-DP were plated on 24-well plates and allowed to attach for 24 h. Cellular medium was collected and PGE_2_ and PGI_2_ concentration analyzed by enzyme immunoassay (for PGE_2_; Cayman Chemical, MI, USA. For PGI_2_; Quantikine, R&D System, Madrid, Spain) following the manufacturer’s instructions. Data were expressed as pg PGE_2_ or PGI_2_ per mg of total protein.

### Statistical analysis

Data are reported as mean and standard error of the mean (SEM) for continuous variables. Comparison and correlation analyses were performed with Student T-test and Pearson coefficient correlation or the correspondent non-parametric tests. Analysis of variance (ANOVA) was used to test for differences in means across treatment groups, and with significant differences a post hoc HSD-Tukey test was used to compare specific treatment groups. Differences with a P value of less than 0.05 were considered statistically significant. Analyses were performed with R version 2.0.1.

## Competing interests

Santiago Redondo has given conferences and received honoraria from Gilead and Merck-Sharp & Dome. The authors declare that they have no competing interests.

## Authors’ contributions

AG-M, ER and SR performed the experiments. AG-M, ER, SR and MC participated in data analysis. AG-M, MC, FR and ER collected the clinical data. AG-M, ER, TT and SR wrote the first draft of the manuscript. All patients contributed in the final version. All authors read and approved the final manuscript.
